# A Computational Approach to Elucidate the Interactions of Chemicals From *Artemisia annua* Targeted Toward SARS-CoV-2 Main Protease Inhibition for COVID-19 Treatment

**DOI:** 10.3389/fmed.2022.907583

**Published:** 2022-06-15

**Authors:** Titilayo Omolara Johnson, Abayomi Emmanuel Adegboyega, Oluwafemi Adeleke Ojo, Amina Jega Yusuf, Opeyemi Iwaloye, Chinenye Jane Ugwah-Oguejiofor, Rita Onyekachukwu Asomadu, Ifeoma Felicia Chukwuma, Stephen Adakole Ejembi, Emmanuel Ike Ugwuja, Saqer S. Alotaibi, Sarah M. Albogami, Gaber El-Saber Batiha, Bodour S. Rajab, Carlos Adam Conte-Junior

**Affiliations:** ^1^Department of Biochemistry, Faculty of Basic Medical Sciences, University of Jos, Jos, Nigeria; ^2^Jaris Computational Biology Centre, Jos, Nigeria; ^3^Phytomedicine, Molecular Toxicology, and Computational Biochemistry Research Group, Department of Biochemistry, Bowen University, Iwo, Nigeria; ^4^Department of Pharmaceutical and Medicinal Chemistry, Faculty of Pharmaceutical Sciences Usmanu Danfodiyo University, Sokoto, Nigeria; ^5^Bioinformatics and Molecular Biology Unit, Department of Biochemistry, Federal University of Technology, Akure, Nigeria; ^6^Department of Pharmacology and Toxicology, Faculty of Pharmaceutical Sciences, Usmanu Danfodiyo University, Sokoto, Nigeria; ^7^Department of Biochemistry, Faculty of Biological Sciences, University of Nigeria, Nsukka, Nigeria; ^8^Department of Biochemistry, Faculty of Science, Ebonyi State University, Abakaliki, Nigeria; ^9^Department of Biotechnology, College of Science, Taif University, Taif, Saudi Arabia; ^10^Department of Pharmacology and Therapeutics, Faculty of Veterinary Medicine, Damanhour University, Damanhour, Egypt; ^11^Laboratory Medicine Department, Faculty of Applied Medical Sciences, Umm Al-Qura University, Makkah, Saudi Arabia; ^12^Technological Development Support Laboratory (LADETEC), Center for Food Analysis (NAL), Federal University of Rio de Janeiro (UFRJ), Rio de Janeiro, Brazil

**Keywords:** coronavirus, SARS-CoV-2, *Artemisia annua*, main protease (M^pro^), rhamnocitrin, isokaempferide, kaempferol

## Abstract

The inhibitory potential of *Artemisia annua*, a well-known antimalarial herb, against several viruses, including the coronavirus, is increasingly gaining recognition. The plant extract has shown significant activity against both the Severe Acute Respiratory Syndrome Coronavirus (SARS-CoV) and the novel SARS-CoV-2 that is currently ravaging the world. It is therefore necessary to evaluate individual chemicals of the plant for inhibitory potential against SARS-CoV-2 for the purpose of designing drugs for the treatment of COVID-19. In this study, we employed computational techniques comprising molecular docking, binding free energy calculations, pharmacophore modeling, induced-fit docking, molecular dynamics simulation, and ADMET predictions to identify potential inhibitors of the SARS-CoV-2 main protease (M^pro^) from 168 bioactive compounds of *Artemisia annua*. Rhamnocitrin, isokaempferide, kaempferol, quercimeritrin, apigenin, penduletin, isoquercitrin, astragalin, luteolin-7-glucoside, and isorhamnetin were ranked the highest, with docking scores ranging from −7.84 to −7.15 kcal/mol compared with the −6.59 kcal/mol demonstrated by the standard ligand. Rhamnocitrin, Isokaempferide, and kaempferol, like the standard ligand, interacted with important active site amino acid residues like HIS 41, CYS 145, ASN 142, and GLU 166, among others. Rhamnocitrin demonstrated good stability in the active site of the protein as there were no significant conformational changes during the simulation process. These compounds also possess acceptable druglike properties and a good safety profile. Hence, they could be considered for experimental studies and further development of drugs against COVID-19.

## Introduction

For quite some time, the COVID-19 outbreak, caused by the severe acute respiratory syndrome-coronavirus 2 (SARS-CoV-2) has had a negative impact on the entire world. The disease began in Wuhan (Hubei, China) in December 2019 (1, 2), spread to the rest of the world, and was declared a pandemic by the World Health Organization (WHO) (3). They are the biggest known RNA infections comprising positive single-stranded RNA and are connected with serious respiratory illnesses (4, 5). The ailment's side effects include, but are not limited to, fever, dry cough, sore throat, and difficulty breathing. Over 468 million reported cases and over 6 million deaths had been documented by the WHO as of March 20, 2022 (6). Although seeing so many vaccinations proven and continuing into development is really exciting, research into how well vaccines protect not only against sickness but also against infection and transmission is still ongoing (7). And, while vaccines remain the most critical tool for containing the pandemic, new medicines are still desperately needed to treat people who cannot or do not want to get vaccines, whose immune systems do not fully respond to vaccination, or who contract breakthrough infections (8).

Developing conventional medications and pipelines can be time-consuming, costly, and sometimes associated with high clinical disappointment (9, 10). Nevertheless, medicinal plants have been explored as a rich source of effective bioactive agents against numerous viral infections (11, 12) and several antiviral plant products are suggested to be active against the SARS-CoV-2 M^pro^ protease (13–16). *Artemisia annua* is a renowned medicinal plant belonging to the Asteraceae family (Compositae) (17, 18) and it is generally known as sweet wormwood or Qinghao (19, 20). The identification of the plant and its artemisinin component constituted a major breakthrough in the fight against malaria. Other pharmacological activities have since then been associated with the plant. These include analgesic, anti-inflammatory, antioxidant, immunomodulatory, antibacterial, anticancer, and antiviral activities (21, 22), among others.

*Artemisia annua* tea is known to be effective against several viral infections, including herpes simplex virus, dengue virus, human immunodeficiency virus (HIV), and coronavirus. The plant extract showed significant activity against the SARS coronavirus that occurred in 2002, and it was selected for its significant inhibitory effect among 200 Chinese medicinal herbs screened for antiviral activities against SARS-CoV (23). Some studies have also reported the *in vitro* inhibitory activity of *Artemisia annua* against SARS-CoV-2 and most of these studies have focused mainly on the artemisinin component and its derivatives (24, 25). It is, however, necessary to explore other known bioactive compounds of the plant, including the non-artemisinin components, to fully maximize the anti-SARS-CoV-2 potential of the plant in the development of drugs against COVID-19. Over the years, many bioactive phytochemicals, such as terpenoids, tannins, coumarins, essential oils, bioflavonoids, and polyphenols, have been isolated and characterized from different parts of *Artemisia annua* (22) and some have been found to possess antiviral activity (26–29). Identification of anti-viral compounds of *Artemisia annua* with more potent SARS-CoV-2 inhibitory activity could therefore help provide an excellent therapeutic alternative against COVID-19.

The discovery of novel therapeutics has advanced in the last two decades toward the use of ground-breaking complementary approaches, such as computational procedures (30). In this manner, bioinformatic devices have been widely used in many intriguing investigations and as of late, for SARS-CoV-2 drug research (31–34). Molecular docking, molecular dynamics simulation, pharmacophore modeling, and ADMET (Absorption, Distribution, Metabolism, Excretion, and Toxicity) studies are some of the major computational methods that offer great applicability in a short period of time (35). The molecular docking technique assists in predicting the binding affinity and inhibitory potential of a test compound (ligand) against a target protein whose modification can produce a therapeutic benefit (36). A well-recognized molecular target for the development of anti-SARS-CoV-2 drugs is the coronavirus main protease (M^pro^) or 3-chymotrypsin-like protease (3CL^pro^), which plays a vital role in viral replication by the processing of the polyproteins that are translated from the viral RNA (37). When SARS-CoV-2 infects a host, replicase polyproteins 1a and 1ab are produced, resulting in a variety of functional subunits required for viral replication. This is done by two viral proteases, one of which is the M^pro^, hydrolyzing the polyproteins at specified sites. Inhibition of this enzyme has been viewed as a crucial approach for SARS-CoV-2 therapeutic intervention (37, 38).

*A. annua* and its artemisinin component have been found in recent studies to inhibit the enzymatic activity of 3CL^pro^ (24, 25). In 2005, the first study indicating *A. annua* as an anti-SARS-CoV agent was reported. It was discovered that *A. annua* was one of four herbs that effectively suppressed the *in vitro* activity of the SARS-CoV (strain BJ001) in a dose-dependent manner (23, 24). However, the mechanism of antiviral effects was not investigated in that study. In a computational study, 33 compounds, including natural products, were tested as potential SARS-CoV-2 3CLpro inhibitors. Artemisinin was one of the substances that interacted with the active binding sites of 3CL^pro^ (24, 39), but it was not the most effective inhibitor. Another computer simulation explored the possibility of 24 natural compounds, 22 US FDA-approved pharmaceuticals, and 16 antimalarial drugs, including artemisinin derivatives, as SARS-CoV-2 3CL^pro^ inhibitors (24, 40). In comparison to lopinavir, amodiaquine, theaflavin digallate, chloroquine, and quinine; artemisinin derivatives (artenimol, artesunate, and artemether) had lower docking scores (24, 40). However, it appears that many of the non-artemisinin components of *A. annua* have not been thoroughly investigated for their M^pro^ inhibitory capabilities. As a result, the current study investigated the SARS-CoV-2 M^pro^ inhibiting potential of 168 phytocompounds from *Artemisia annua* using a detailed computational analysis that included molecular docking, binding free energy calculations, pharmacophore modeling, induced-fit docking, molecular dynamics simulation, and ADMET predictions.

## Materials and Methods

### Protein Preparation

Preparation of protein was carried out as previously described (41, 42). The crystal structure of SARS-CoV-2 main protease (M^pro^), also known as 3-Chymotypsinlike protease (3CLpro) with PDB ID: 7C6U, was gotten from the Protein Data Bank (PDB) repository. Using Glide (Schrödinger Suite 2020-3), the protein structure obtained was prepared *via* the protein preparation wizard panel. During the preparation processes, hydrogen was added, bond orders were allocated, disulfide bonds were generated, the side chains and loops that were missing were replaced using prime. Water molecules located outside 3.0 Å of the heteroatoms were removed, and the protein structure was minimized using OPLS3e and optimized using PROPKA.

### Generation of Receptor Grid

The receptor grid was created to outline the position and size of the protein's active site for ligand docking. This was done using the receptor grid generation tool of Schrödinger Maestro 12.5. The position of the co-crystalized ligand (K36) in the active site of the protein was used as the scoring grid (41, 42).

### Ligand Preparation

One hundred and sixty-eight (168) bioactive compounds of *Artemisia annua* obtained from Dr. Duke's Phytochemical and Ethnobotanical Databases and the standard ligand ((1S,2S)-2-({N-[(benzyloxy)carbonyl]-L-leucyl}amino)-1-hydroxy-3-[(3S)-2-oxopyrrolidin-3-yl]propane-1-sulfonic acid or K36) were prepared using the Ligprep panel of Maestro 12.5, Schrödinger Suite 2020-3, and the protocol is as previously described (32). The ligands were prepared to obtain low-energy 3D structures with appropriate chiralities. The ionization state for each ligand structure was generated at a physiological pH of 7.2 ± 0.2. Stereoisomers of each ligand were computed by retaining specified chiralities while others were varied.

### Protein-Ligand Docking

The molecular docking analysis was carried out using the Glide-Ligand Docking panel of Maestro 12.5 on Schrödinger Suite 2020-3. The prepared ligands and the receptor grid file were imported into the work space of Maestro. Using standard precision (SP) docking, the ligands were docked into the binding pocket of the target protein. The vdW radius scaling factor was scaled at 0.80 with a partial charge cut-off of 0.15 for ligand atoms, and the ligand sampling method was set to be flexible (32).

### Binding Free Energy Calculation

The MM-GBSA Prime panel of the Schrödinger Suite 2020-3 was used to estimate the binding free energy of the receptor-ligand complex. Prior to that, the ligands were prepared using ligprep, and the proteins were prepared using the protein preparation wizard, as detailed earlier (42). Sitemap was used to identify the active sites of the proteins, and glide extra precision (XP) docking was used to dock the chemicals with proteins. The MM-GBSA technology of Prime was then utilized to determine the binding free energy for ligand-protein complexes (42). The OPLS3 force field was chosen, and the continuum solvent model was VSGB. The default settings were chosen for the other options.

### Receptor-Ligand Complex Pharmacophore Modeling

The PHASE module of the Schrödinger Suite 2020-3 was used to generate an auto/e-pharmacophore model as previously described (43). The process was set to generate a maximum of 7 features at a minimum feature–feature distance of 2.00, and the minimum distance between features of the same type was set at 4.00. Donors were set as vectors.

### Induced Fit Docking

The molecular interaction of rhamnocitrin with the receptor (M^pro^) was elucidated using Maestro 12.5's induced fit docking panel according to the protocol described in Schrödinger (44). The Induced Fit Docking methodology aims to improve ligand docking in cases when the receptor is thought to adjust considerably to the presence of the ligand. The method begins with a constrained receptor minimization and then employs a softening potential to achieve the ligands' first glide docking. Twenty sets of the docked poses were passed on to Prime for a refinement step. The best receptor structures for each ligand were submitted back to Glide for redocking after prime side-chain prediction and minimization. The extended sampling approach automates receptor construction by selecting residues to trim and calculating atom-specific van der Waals scaling factors based on solvent-accessible surface areas, B-factors, salt bridges, and rotamer searches. The initial docking stage generates a huge number of poses, which are then grouped and filtered to provide up to 80 poses per ligand, which are then passed on to the prime step. The Glide SP was used to score the final docked poses.

### Molecular Dynamics Simulation

The Simulation module of the Molecular Operating Environment MOE 2019.01 software was used to perform molecular dynamics simulations (45). To obtain the stable conformer of the protein-ligand complex in an R-Field implicit solvation system, the protein and protein-ligand complex were protonated, energy was minimized, and parameterized with the AMBER 10: EHT force field at various times. The simulations were carried out in three steps. The molecular system was first heated to 310 K (37°C). This was followed by an equilibration step of 100 picoseconds at 310 K (37°C). The molecular system's trajectory was then generated for 1,000 picoseconds at 310 K using the Nose–Poincare–Andersen (NPA) algorithm (the time step of each simulation was set to 0.02 picoseconds). Visualizations and data analysis were carried out using VMD software and Bio3D on the Galaxy Europe platform. The system's essential dynamics were modeled using the principal components analysis (PCA) (46). The simulation data set was reduced to a few key components that describe the directions with the largest variance. The key structural variations within the ensemble of protein structures were captured by ranking the principal components as eigenvectors depending on the variance. An eigenvalue rank plot was created to display the fraction of variance attributable to each primary component. This was followed by structural clustering based on the resulting principal components and residue-wise loadings to see how much each residue contributed to the first two principal components.

### ADMET Profiling

The ADMET properties of the selected test compounds were determined using *in silico* predictive models. The SwissADME server was used to determine the ADME properties of the compounds, which include: lipophilicity (Log P), water solubility (ESOL Log S), drug-likeness based on the Lipinski rule and bioavailability score; and pharmacokinetics based on gastrointestinal (GI) absorption, blood brain barrier (BBB) permeant, permeability glycoprotein (Pgp) binding and Cytochrome P450 (CYP) inhibition (47). The ProTox-II online server was used to predict the acute toxicity class, LD_50_, hepatotoxicity, carcinogenicity, mutagenicity, cytotoxicity, and immunotoxicity of the compounds (48).

## Results

### Molecular Docking Analysis

**Figure 1:**
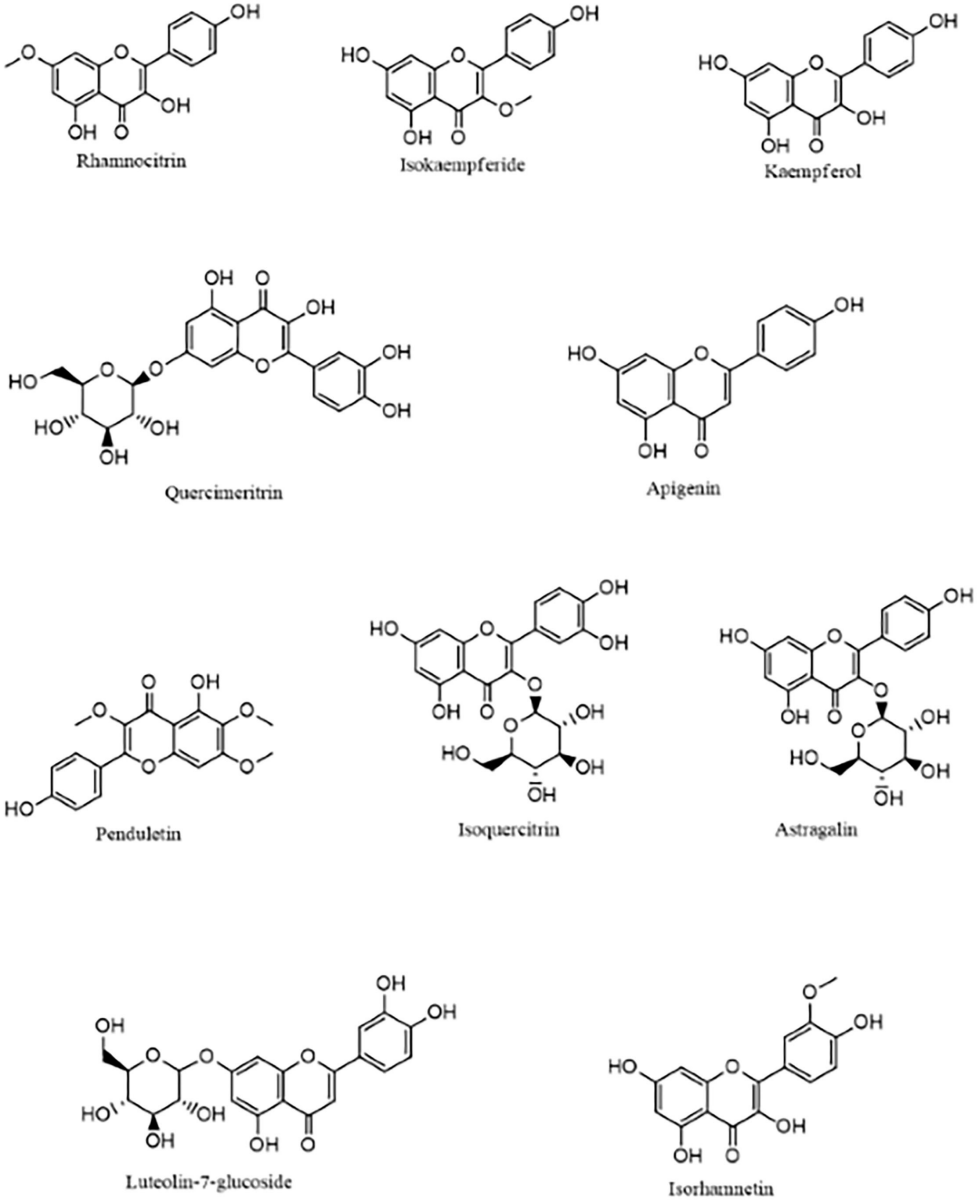
The 10 top-scoring compounds of *Artemisia annua* against SARS-CoV-2 main protease.

The molecular docking analysis showed that the compounds of *Artemisia annua* possess varying levels of binding affinities for the SARS-CoV-2 main protease, the top 10 being Rhamnocitrin (7-Methylkaempferol), Isokaempferide (3-Methylkaempferol), Kaempferol, Quercimeritrin, Apigenin, Penduletin, Isoquercitrin, Astragalin, Luteolin-7-glucoside and Isorhamnetin (Figure 1). The binding affinities of these compounds are higher than those of the standard ligand, which is −6.59 kcal/mol. Rhamnocitrin scored highest, with a docking score of −7.83 kcal/mol followed by Isokaempferide with −7.81 kcal/mol, and Kaempferol with −7.65 kcal/mol (Table 1). The entire list of the 168 compounds with their docking scores (kcal/mol) and binding free energy (ΔG _Bind_) MM-GBSA against the SARS-CoV-2 main protease can be found in Supplementary Table 1.

**Table 1:** Names and docking scores (kcal/mol) of the 10 top-scoring compounds of *Artemisia annua* against SARS-CoV-2 main protease.

**Compound**	**Synonym**	**Structural class**	**Docking score (kcal/mol)**
Rhamnocitrin	7-Methylkaempferol	Flavonoid	−7.83
Isokaempferide	3-Methylkaempferol	Flavonoid	−7.81
Kaempferol	3,4′,5,7-Tetrahydroxyflavone	Flavonoid	−7.65
Quercimeritrin	Quercetin 7-glucoside	Flavonoid	−7.55
Apigenin	4′,5,7-Trihydroxyflavone	Flavonoid	−7.49
Penduletin	5,4′-Dihydroxy-3,6,7-trimethoxyflavone	Flavonoid	−7.38
Isoquercitrin	Quercetin 3-glucoside	Flavonoid	−7.33
Astragalin	kaempferol-3-glucoside	Flavonoid	−7.23
Luteolin-7-glucoside	Flavopurposide	Flavonoid	−7.16
Isorhamnetin	3-Methylquercetin	Flavonoid	−7.15
K36	SCHEMBL21114829	Standard ligand^*^	−6.59

* *K36 - (1S,2S)-2-({N-[(benzyloxy)carbonyl]-L-leucyl}amino)-1-hydroxy-3-[(3S)-2-oxopyrrolidin-3-yl]propane-1-sulfonic acid*.

**Figure 2:**
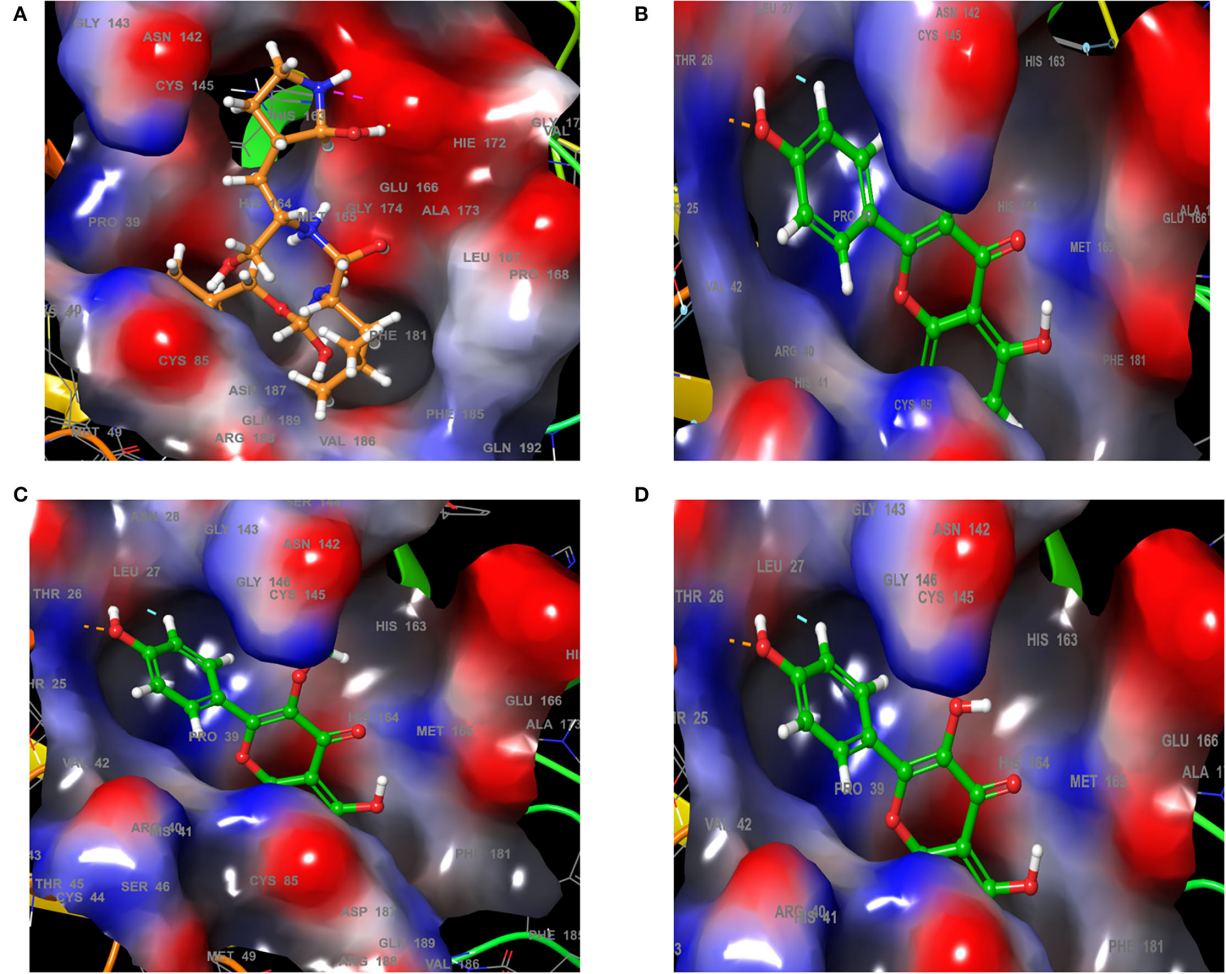
The 3D view of the molecular interaction of **(A)** K36, **(B)** Rhamnocitrin, **(C)** Isokaempferide, and **(D)** Kaempferol with SARS-CoV-2 main protease.

**Figure 3:**
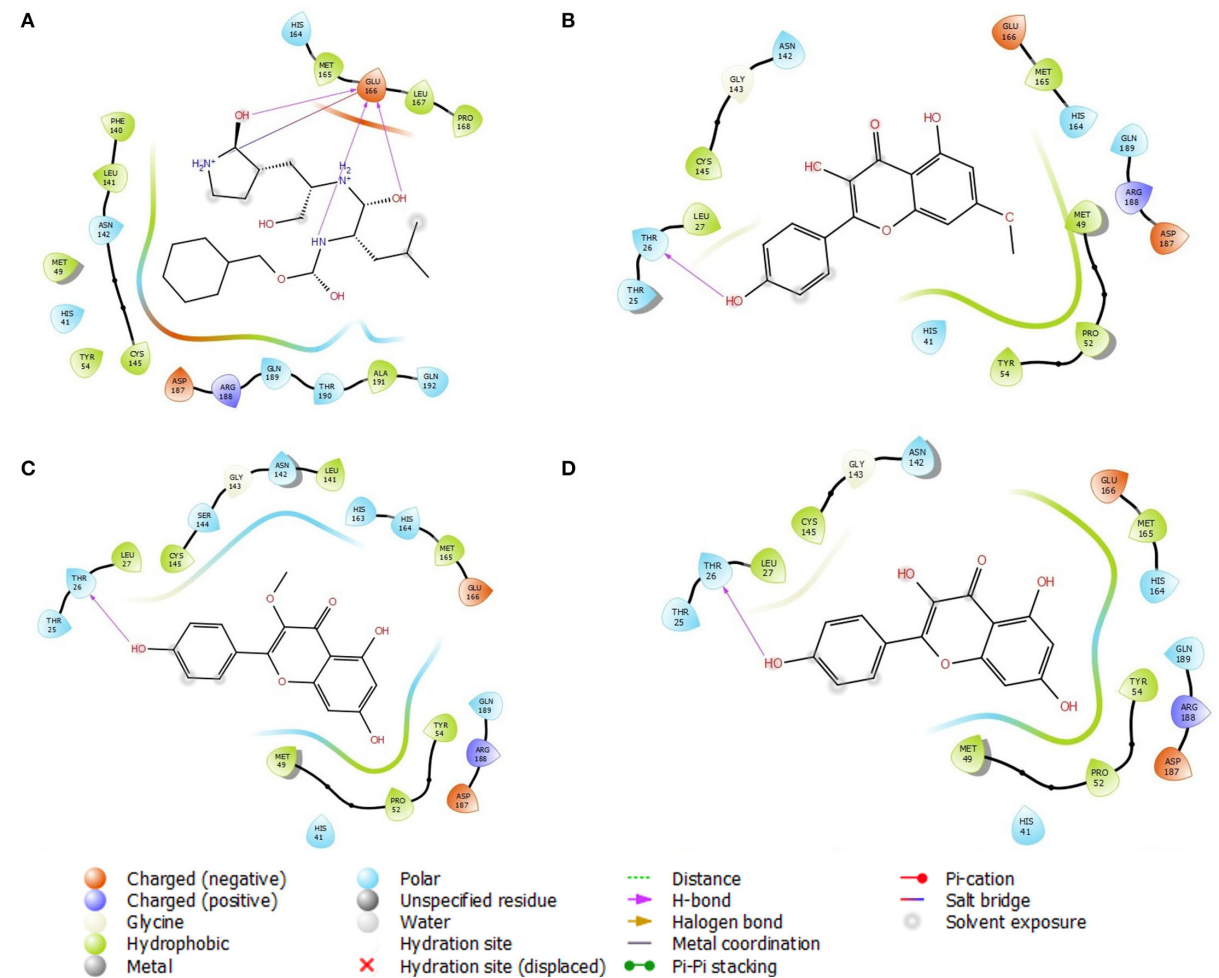
The 2D view of the molecular interaction of **(A)** K36, **(B)** Rhamnocitrin, **(C)** Isokaempferide, and **(D)** Kaempferol with SARS-CoV-2 main protease.

Analysis of the 3D and 2D structures of the complexes formed by the three top-scoring compounds (Rhamnocitrin, Isokaempferide, and Kaempferol) with SARS-CoV-2 M^pro^ showed that the compounds occupied the active site of the enzyme (Figures 2, 3). The three compounds and the standard ligand (K36) made contact with important active site amino acid residues like HIS 41, ASN 142, CYS 145, and GLU 166. K36 formed three hydrogen bonds with GLU 166 and hydrophobic interactions with MET 49, TYR 54, PHE 140, LEU 141, CYS 145, MET 165, LEU 167, PRO 168, and ALA 191. Rhamnocitrin, Isokaempferide, and Kaempferol formed hydrogen bond with THR 26 and hydrophobic interactions with LEU 27, MET 49, PRO 52, TYR 54, CYS 145, and MET 165.

### Binding Free Energy Calculation

**Figure 4:**
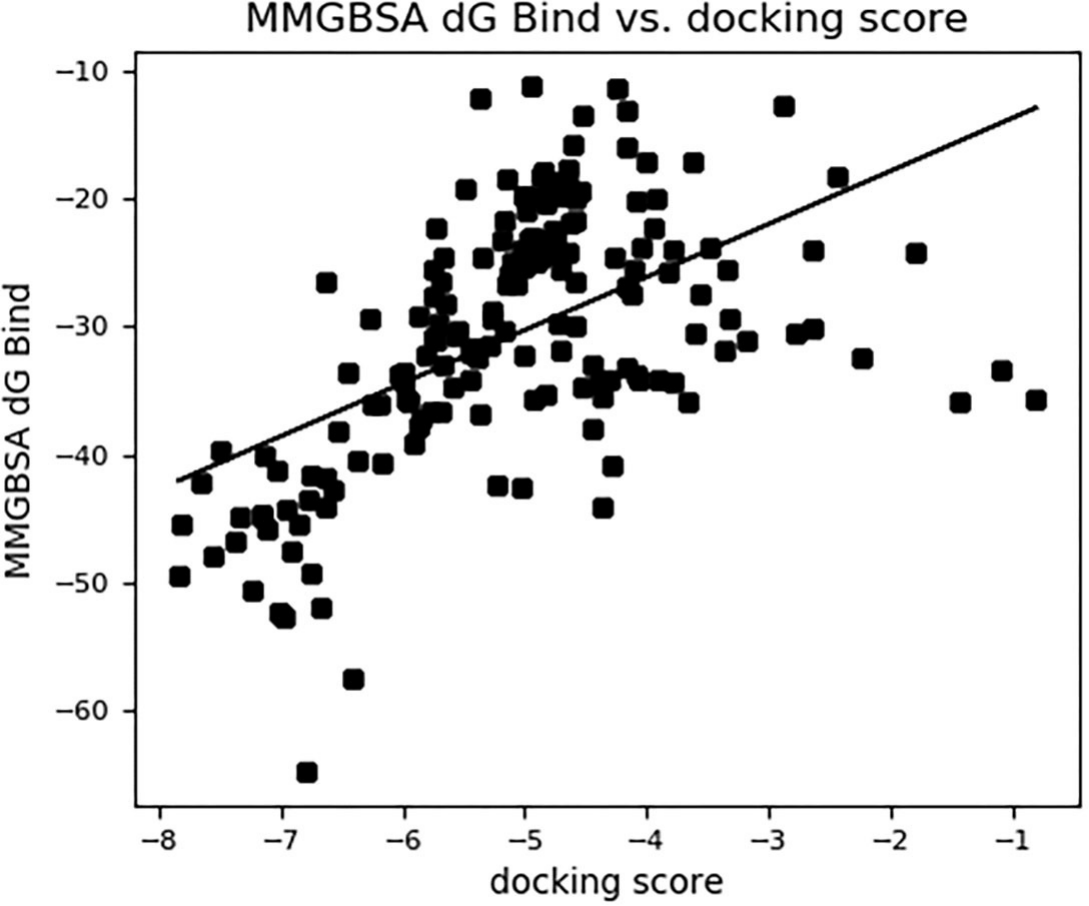
The binding free energy MM-GBSA (Δ*G*_bind_) vs. the docking score (kcal/mol) of 168 compounds of *Artemisia annua* against SARS-CoV-2 main protease.

The binding free energies of the top 10 compounds are shown in Table 2, Figure 4 shows the scatter plot of the binding free energy (ΔG _Bind_) vs. docking score (kcal/mol) of the 168 phytochemical compounds of *Artemisia annua* against SARS-CoV-2 M^pro^. This post-docking analysis showed that the 10 top-scoring compounds of *Artemisia annua* had binding free energy values ranging between −39.67 and −50.61 kcal/mol. Most of the points are, however, close to the regression line of the scatter plot.

**Table 2:** The binding free energy (Δ*G*_bind_) MM-GBSA of 10 top-scoring compounds of *Artemisia annua* against SARS-CoV-2 main protease.

**Compound**	**ΔG _**Bind**_**	**ΔG _**Bind**_**	**ΔG _**Bind**_**	**ΔG _**Bind**_**	**ΔG _**Bind**_**	**ΔG _**Bind**_**	**ΔG _**Bind**_**	**ΔG _**Bind**_**
		**Coulomb**	**Covalent**	**Hbond**	**Lipo**	**Packing**	**Solv GB**	**vdW**
Rhamnocitrin	−49.53	−14.04	4.05	−1.48	−7.8	−4.39	12.69	−38.57
Isokaempferide	−45.34	−11.95	4.72	−1.35	−6.27	−4.66	14.89	−40.72
Kaempferol	−42.09	−12.94	3.53	−1.36	−5.68	−4.83	16.46	−37.28
Quercimeritrin	−47.93	−35.28	5.34	−4.07	−9.88	−3.31	35.39	−36.12
Apigenin	−39.67	−9.98	4.48	−1.35	−5.09	−4.88	13.62	−36.47
Penduletin	−46.7	−24.39	3.1	−1.73	−11.02	−3.18	25.17	−34.66
Isoquercitrin	−44.77	−9.6	2.05	−2.87	−10.85	−2.67	28.01	−48.84
Astragalin	−50.61	−11.26	2.98	−2.26	−11.52	−2.54	23.26	−49.26
Luteolin-7-glucoside	−44.92	−36.17	5.52	−2.87	−9.78	−2.85	37.88	−36.64
Isorhamnetin	−44.74	−13.02	3.87	−1.38	−6.31	−4.74	17.57	−40.73

### Pharmacophore Modeling of the Top-Three *A. annua* Compounds

**Figure 5:**
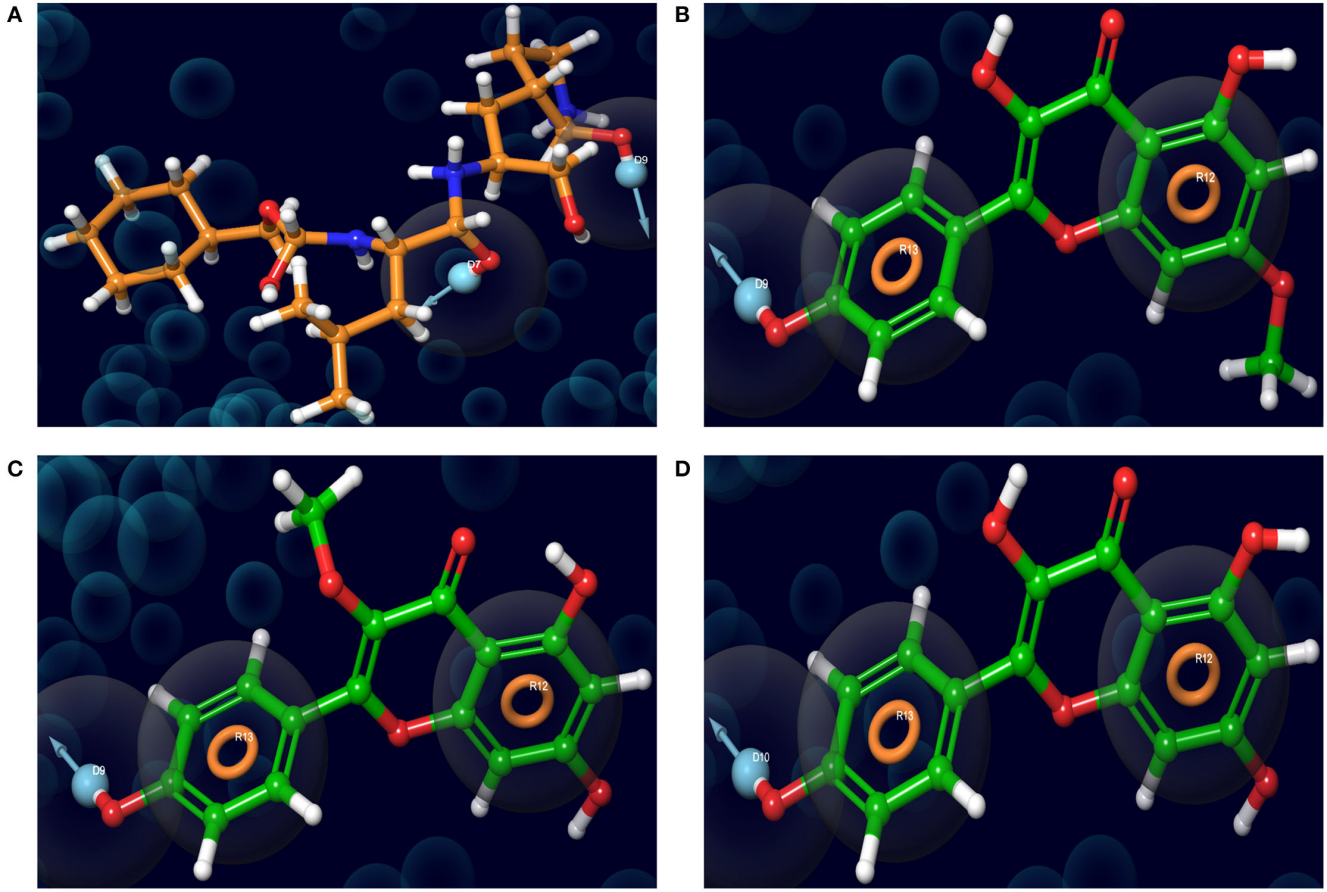
The receptor-ligand complex pharmacophore models of **(A)** K36, **(B)** 7-Methylkaempferol, **(C)** 3-Methylkaempferol, and **(D)** Kaempferol on SARS-CoV-2 main protease.

The pharmacophore models of the standard ligand, Rhamnocitrin, Isokaempferide, and Kaempferol on M^pro^ are shown in Figure 5. Two hydrogen bond donors contributed to the binding of the standard ligand to the enzyme, while two aromatic rings and one hydrogen bond donor were involved in the molecular interactions of the three test compounds with the enzyme.

### Molecular Modeling of the Biological Interactions of the Top-Scoring Compound of *A. annua* With SARS-CoV-2 Main Protease

#### Induced-Fit Docking

**Figure 6:**
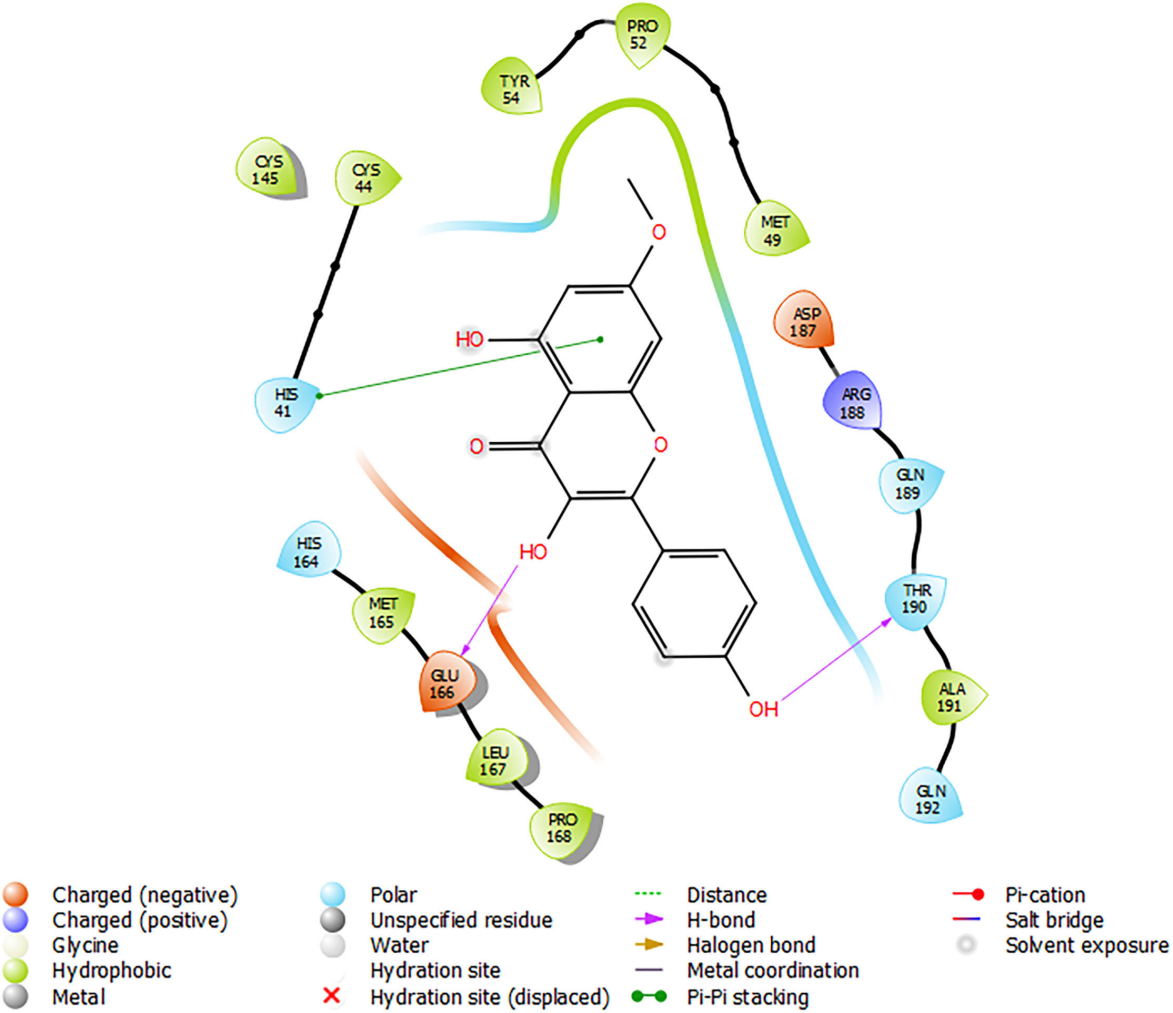
Molecular interactions of Rhamnocitrin with SARS-CoV-2 main protease from the induced fit docking.

Figure 6 shows the molecular interactions of Rhamnocitrin (the most promising *A. annua* compound) in the flexible active site of SARS-CoV-2 M^pro^ following the induced fit docking. The compound was shown to form a pi–pi stacking interaction with HIS 41, hydrogen bonds with GLU 166 and THR 190, and hydrophobic contacts with CYS 44, MET 49, PRO 52, TYR 54, CYS 145, MET 165, LEU 167, PRO 168, ALA 191.

#### Molecular Dynamics Simulation

**Figure 7:**
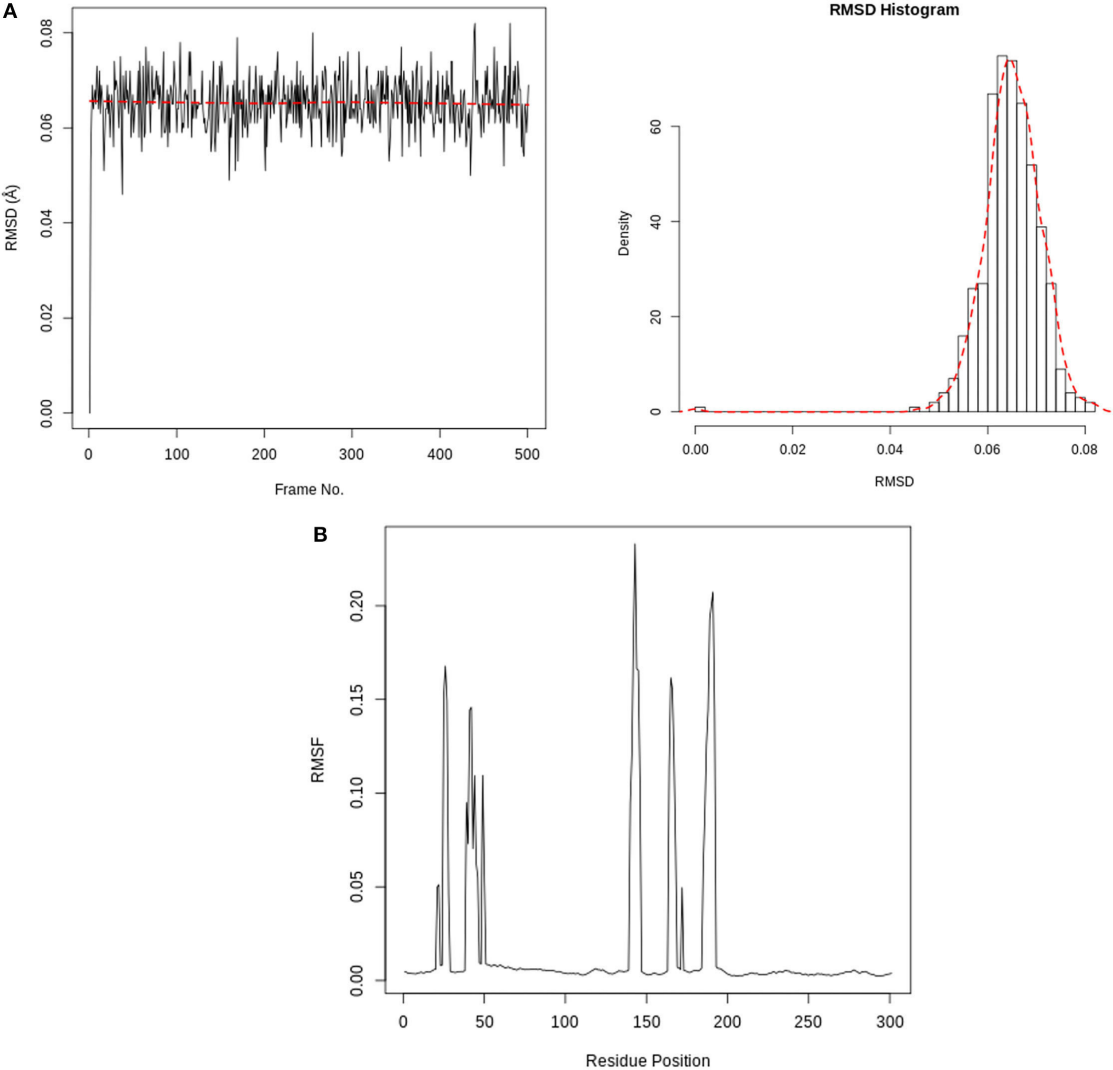
**(A)** RMSD time series and histogram for Rhamnocitrin in the active site of SARS-CoV-2 main protease. **(B)** SARS-CoV-2 main protease RMSF(Å) vs. the residue position.

**Figure 8:**
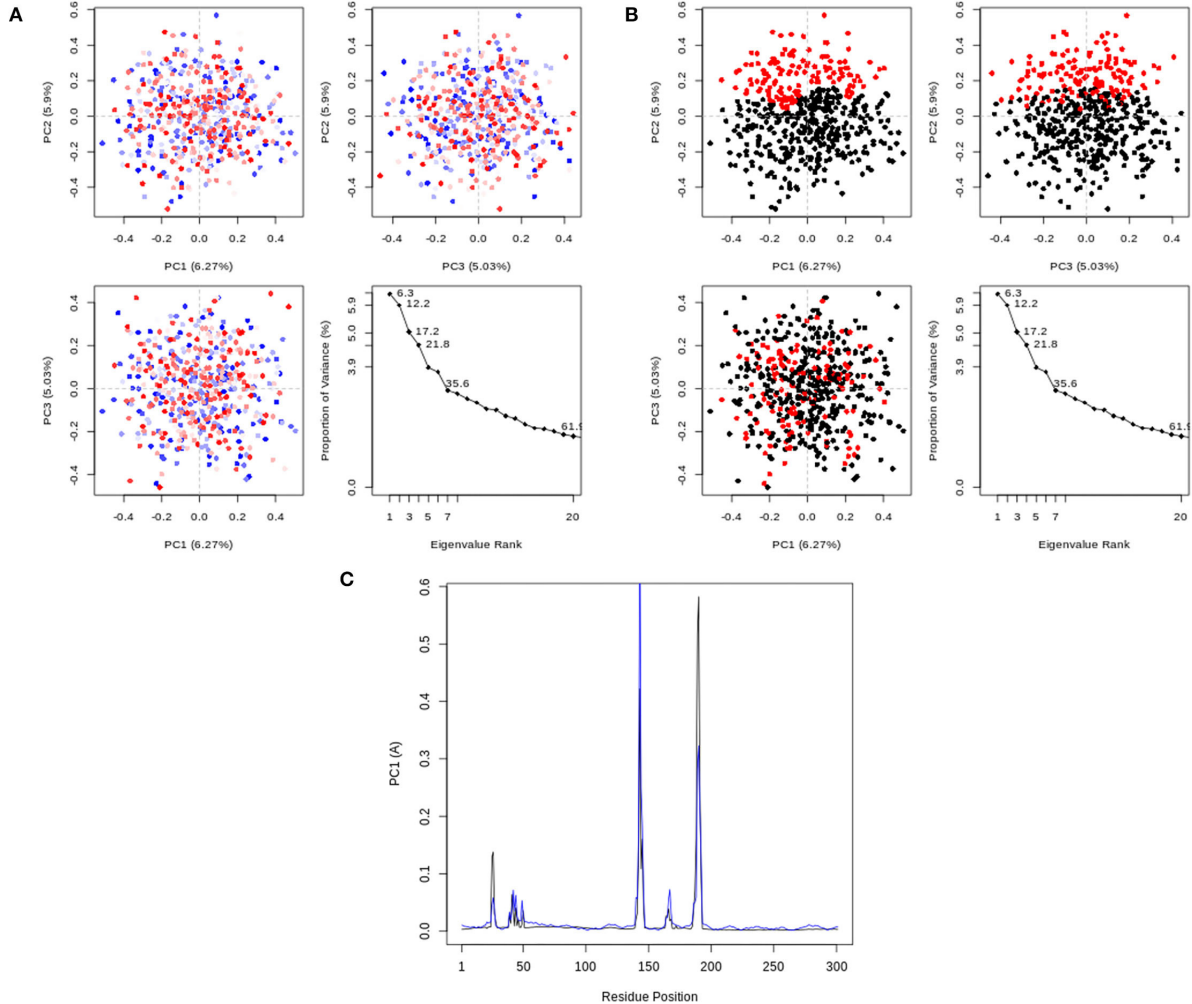
PCA results, comprising graphs of PC2 vs. PC1, PC2 vs. PC3, PC3 vs. PC1, and an eigenvalue rank plot with the cumulative variance annotated for each data point. **(A)** PCA plots colored from blue to red in order of time; **(B)** PCA Plots showing two different clusters colored black and red. **(C)** Residue-wise loadings for PC1 (black) and PC2 (blue).

Figure 7 depicts the RMSD and RMSF plots, as well as the RMSF histogram. The RMSD maintains a stable fluctuation between 0.06 and 0.08 Å after an initial rise from 0.00 Å. The RMSF shows fluctuations at various positions, the major ones being around positions 143 and 190. Figure 8 illustrates the PCA outputs, which comprise graphs of PC2 vs. PC1, PC2 vs. PC3, and PC3 vs. PC1, an eigenvalue rank plot (A and B), and the result of residue-wise loadings. The cumulative variance is labeled for each data point in the eigenvalue plot. According to the results, the first principal component (PC1) accounts for 6.3% of the total variance, and the first three principal components account for 17.2% of the variance. A continuous color scale from blue to white to red was attained along the PC planes (A). The trajectory snapshots were separated into two different clusters of the colors black and red through the top three PC1, PC2, and PC3 spaces (B). The result of residue-wise loadings for PC1 (black) and PC2 (blue) shows high peaks on residues 143 and 190.

### ADMET Profile

According to the results displayed in Table 3, the molecular weights of rhamnocitrin and isokaempferide are 300.26 while that of kaempferol is 286.24. The log S value is −3.51 for rhamnocitrin and isokaempferide, and −3.31 for kaempferol. The three compounds have log *p*-values of 1.98, 1.94, and 1.58, respectively, and have zero Lipinski violations, a bioavailability score of 0.55, and a high gastrointestinal (GI) absorption potential. None of the compounds is a permeability glycoprotein (Pgp) substrate or a blood brain barrier (BBB) permeant. In terms of cytochrome P450 inhibitory potencies, the three compounds are potential CYP1A2, CYP2D6, and CYP3A4 inhibitors. The three chemicals are classified as oral toxicity class 5 by ProTox II, with isokaempferide and kaempferol having an LD_50_ of 3,919 mg/kg and rhamnocitrin having an LD_50_ of 4,000 mg/kg. There is no indication of hepatotoxicity, carcinogenicity, mutagenicity, cytotoxicity, or immunotoxicity in any of them (Table 4).

**Table 3:** Druglikeness and pharmacokinetics prediction of the three top-scoring *Artemisia annua* compounds.

	**Rhamnocitrin**	**Isokaempferide**	**Kaempferol**
Molecular weight	300.26	300.26	286.24
ESOL log S	−3.51	−3.51	−3.31
Solubility class	Soluble	Soluble	Soluble
Mean log P	1.98	1.94	1.58
Lipinski violations	0	0	0
Bioavailability score	0.55	0.55	0.55
GI absorption	High	High	High
BBB permeant	No	No	No
Pgp substrate	No	No	No
CYP1A2 inhibitor	Yes	Yes	Yes
CYP2C19 inhibitor	No	No	No
CYP2C9 inhibitor	No	No	No
CYP2D6 inhibitor	Yes	Yes	Yes
CYP3A4 inhibitor	Yes	Yes	Yes

**Table 4:** Toxicity profile of the three top-scoring *Artemisia annua* compounds.

	**Rhamnocitrin**	**Isokaempferide**	**Kaempferol**
LD_50_ (mg/kg)	4,000	3,919	3,919
Toxicity class	5	5	5
Hepatotoxicity	Inactive	Inactive	Inactive
Carcinogenicity	Inactive	Inactive	Inactive
Immunotoxicity	Inactive	Inactive	Inactive
Mutagenicity	Inactive	Inactive	Inactive
Cytotoxicity	Inactive	Inactive	Inactive

## Discussion

The main protease (M^pro^) is an indispensable enzyme responsible for viral replication and it is among the most characterized drug targets of coronaviruses (37). Upon host infection by SARS-CoV-2, replicase polyproteins 1a and 1ab are synthesized to produce various functional subunits essential for viral replication. This is accomplished through the site-specific hydrolysis of polyproteins by two viral proteases, one of which is M^pro^ (38). Inhibition of this enzyme has been recognized to be an important strategy for therapeutic intervention against SARS-CoV-2. In this study, computational techniques were employed to identify possible SARS-CoV-2 inhibitors among the compounds of *Artemisia annua*, an antimalarial plant with reported activity against the SARS-CoVs.

The compounds of *Artemisia annua* showed varying levels of binding affinities for the SARS-CoV-2 main protease in the molecular docking analysis, the highest being −7.83 kcal/mol by 7-methylkaempferol (rhamnocitrin), followed by −7.81 kcal/mol by 3-methylkaempferol (isokaempferide) and −7.65 by Kaempferol. It is worth noting that all the ten top-scoring compounds are flavonoids, which include kaempferol and quercetin derivatives. Kaempferol, a tetrahydroxyflavone with its four hydroxy groups at positions 3, 5, 7, and 4, is a plant-derived aglycone flavonoid commonly found in medicinal herbs, fruits, seeds, and vegetables. This phytochemical has been demonstrated to possess several pharmacological actions, among which are, antioxidant, anti-inflammatory, anticancer, and antiviral activities (49). Kaempferol has been found to display antiviral activity against several viruses, including coronavirus (50, 51). A recent study by Xia et al. identified Kaempferol and Quercetin, along with Luteolin, as the main active ingredients of the Chinese herbal combination (Amygdalus Communis Vas and Ephedra sinica Stapf) used in the treatment of COVID-19 (52). They also reported that these active ingredients demonstrated a good binding affinity for SARS-CoV-2 M^pro^ (52) as substantiated in this study.

The reliability of the molecular docking result was determined by estimating the binding free energy through the Prime MM-GBSA module of Maestro. A favorable binding free energy correlates to a reliable output from a molecular docking study. Binding free energy is an estimation of all intermolecular interactions between the ligand and the molecular target, whereas docking score gives an indication of the binding affinity of the ligand and the target following docking. The binding affinity as represented by the docking score is dependent on the intermolecular interactions between the ligand and the target. A plot of binding free energy vs. docking score gives a picture of the correlation between the results of the docking analysis and the binding free energy. From the scatter plot in Figure 4, most of the points are close to the regression line, which is an indication that the molecular docking result is comparable to a large extent to the binding free energy of the compounds, thereby validating the docking results. This post docking analysis also helps to determine the stability of the receptor-ligand complex (53, 54). The analysis showed that the 10 top-scoring phytochemical constituents of *Artemisia annua* possess rich binding free energy values (−39.67 kcal/mol and −50.61 kcal/mol) toward SARS-CoV-2 main protease. Studies have shown that the lower (more negative) the binding free energy, the more favorable and stable the ligand-bound protein is (42, 55). With this in mind, the ten selected compounds could be said to form favorable complexes with the protein crystal structure of SARS-CoV-2 main protease.

*In silico* pharmacophore modeling of the best three compounds was employed to identify the structural features responsible for their affinity for the target protein. This is to further confirm the compounds' inhibitory potential against the SARS-CoV-2 main protease. Aromatic rings and hydrogen bonds were identified as the structural features responsible for their interactions with the enzyme. The involvement of aromatic rings (like the pi-pi stacking with His 41 discussed below) in addition to hydrogen bond formation, in the interaction of the compounds with the enzyme, might have contributed to the higher binding affinity of the compounds compared with the standard ligand, which depended on hydrogen bond donors only. Aromatic rings are vital residues for molecular interactions and frequently exist in several protein–ligand and protein–protein interactions (43, 56). Owing to their natural existence in amino acid residues like histidine, tryptophan, phenylalanine, and tyrosine, they are considered to be very important for protein stability and molecular recognition processes. Furthermore, aromatic rings are frequently used in drug design due to their role in the improvement of binding affinity and specificity of drug-like molecules (57).

The molecular interactions exhibited by rhamnocitrin, isokaempferide, and kaempferol with the active site amino acid residues of SARS-CoV-2 main protease are an indication of their inhibitory potential and therapeutic benefits against COVID-19. HIS 41 and CYS 145, to which these compounds bind, are known to play very crucial roles at the active site of the SARS coronavirus M^pro^. Previous studies on the crystal structure of the SARS-CoV-2 main protease showed that the protein is composed of three domains: domain I, comprising of residues 10–99, domain II, comprising of residues 100–182 and domain III, comprising of residues 198–303. The active site holds a HIS 41-CYS 145 catalytic dyad in a cleft between domains I and II, with CYS 145 acting as a nucleophile during the first step of the enzymatic process and HIS 41 acting as a base catalyst (58, 59). Also of interest is the interaction of the compounds with GLU 166, which is essential for M^pro^ substrate-induced dimerization required for catalysis, and ASN 142, which forms hydrogen bond with GLU 166 to block the substrate-binding subsite entrance in the monomer. Mutation of GLU 166 is reported to significantly block the substrate-induced dimerization process, thereby preventing enzyme activation (60). Therefore, the interaction of *A. annua* chemicals with these amino acid residues, which are targets of most SARS-CoV-2 main protease inhibitors, makes them potential therapeutic agents against the virus. This is further corroborated by the result of the induced-fit docking of rhamnocitrin against the M^pro^.

Induced-fit docking (IFD) is a method for modeling the conformational changes induced by ligand binding (61). In standard docking analysis, the receptor is held rigid and the ligand is free to move, but in reality, many proteins undergo conformational changes upon ligand binding. These modifications allow the receptor to adjust its binding site to better match the ligand's structure and binding mode (44). In this study, the IFD was used to generate more accurate binding interactions between the most promising compound (rhamnocitrin) and the M^pro^. The result showed important molecular interactions like the pi-pi stacking with His 41, hydrophobic contacts with CYS 145 together with eight other residues, and hydrogen bonding with GLU 166 and THR 190. These interactions may limit the catalytic roles of these key amino acid residues, thus promoting the compound's inhibitory activity.

The interactions between Rhamnocitrin and SARS-CoV-2 main protease, as demonstrated by molecular docking and IFD, were further studied using molecular dynamics (MD) simulation. To check the stability and conformation of the protein and ligand throughout the simulation period, the Root Mean Square Deviation (RMSD) and Root Mean Square Fluctuation (RMSF) were determined. The correlation between statistically relevant conformations (major global motions) recorded during the trajectory was determined using the principal component analysis (PCA) (46). As suggested by the RMSD result (Figure 7), rhamnocitrin was observed to be stable with a single binding mode in the active site of the protein. The RMSD only swings between 0.06 and 0.08 Å which implies that there are no significant conformational changes during the simulation process. The histogram also clearly explains this. For the RMSF, major changes occur around positions 143 and 190, which correspond to flexible loop areas on the protein's surface (Figure 7). According to the eigenvalue rank plot (Figures 8A,B) the first three principal components account for 17.2% of the total variance, and the first principal component (PC1) is responsible for 6.3% of the variance. The result of the PCA study revealed a conformational change in the protein backbone represented by the blue to the white and then to the red color, which was similarly categorized into two coordinate clusters in black (first cluster) and red (second cluster) using the simple clustering in PC subspace. This is in line with the results of the residue-wise loadings (Figure 8C) and RMSF (Figure 7), which revealed major fluctuations around residues 143 and 190. The ability of Rhamnocitrin to achieve and retain a stable conformation within the flexible protein's active site during the simulation is an indication of the stability of the complex, which is a significant advantage for its inhibitory potential against the SARS-CoV-2 main protease.

In spite of the M^pro^ inhibitory potential exhibited by the *Artemisia annua* compounds, the ADMET properties of the compounds are essential factors that will determine their therapeutic effectiveness against COVID-19. *In silico* ADMET prediction is a fast and low-cost approach to determine whether the compounds will be easily absorbable, well-distributed to their target site of action, favorably metabolized, and easily eliminated from the body without leaving toxic side effects (62). The Lipinski filter has been an effective method for screening potential drug candidates for oral drug-likeness based on their molecular weights, hydrogen bond acceptors and donors, and lipophilicity (63). Therefore, compounds like rhamnocitrin, isokaempferide, and kaempferol, with zero Lipinski violations are likely to be orally active, and this is further validated by their oral bioavailability scores. The 0.55% bioavailability score means that these compounds have about a 55% probability of a minimum of 10% oral absorption in rat or human colon carcinoma absorptivity (64). Compounds with 2 or more Lipinski violations and lower bioavailability scores are not likely to be orally active. This is also reflected in the GI absorption potential of the compounds, which is high for the three selected compounds. However, none of the compounds showed BBB permeability. Moreover, the cytochrome P450 inhibitory potentials of rhamnocitrin, isokaempferide, and kaempferol suggest that the compounds may interact with other drugs. This is because certain CYP isoforms metabolize more than half of all medicines, and inhibiting them, which could impair the metabolism of other pharmaceuticals, is a common cause of pharmacokinetics-related drug–drug interactions (47).

It is interesting to note that the toxicity predictions for the compounds appear to be favorable (Table 4). All of the compounds are in the oral toxicity class 5, with LD_50_ values of 3,919 and 4,000 mg/kg, implying that they could be used safely within these dosage limits. Besides that, none of the compounds are inclined to be hepatotoxic, carcinogenic, mutagenic, or cytotoxic, making them relatively safe as potential SARS-CoV-2 therapeutic agents. It is also worth mentioning that this *in silico* investigation is not a perfect duplication of the exact cellular circumstances and normal functioning of an entire organism. Therefore, the selected *A. annua* compounds must be further evaluated using *in vitro* and/or *in vivo* methodologies.

In conclusion, out of 168 bioactive compounds of *Artemisia annua* screened for possible inhibitory activity against SARS-CoV-2 main protease, rhamnocitrin exhibited the highest binding affinity, followed by isokaempferide and kaempferol. The three compounds, like the standard ligand, occupied the active site of M^pro^, where they interacted with important amino acid residues like HIS 41, ASN 142, CYS 145, and GLU 166, among others. Rhamnocitrin was found to be stable, with a single binding mode in the protein's active site throughout the simulation. The three selected compounds possess a favorable ADMET profile and none of them showed the tendency for hepatotoxicity, carcinogenicity, mutagenicity, cytotoxicity, and immunotoxicity. Therefore, these *Artemisia annua* compounds could be considered for experimental studies and further development into drugs for the treatment of SARS-CoV-2.

## Data Availability Statement

The original contributions presented in the study are included in the article/Supplementary Material, further inquiries can be directed to the corresponding author/s.

## Author Contributions

TOJ and AEA spearheaded the project and collaborated with OAO, AJY, OI, CJU-O, ROA, IFC, SAE, and EIU to design the work. TOJ, OAO, AJY, and OI interpreted the results. TOJ, OAO, OI, CJU-O, ROA, and IFC wrote the first draft of the manuscript. The acquisition of funding was the responsibility of SSA, SMA, GE-SB, CAC, BSR, and OAO, who also cooperated on the revision of the paper. The final draft was reviewed and approved by all author.

## Conflict of Interest

The authors declare that the research was conducted in the absence of any commercial or financial relationships that could be construed as a potential conflict of interest.

## Publisher's Note

All claims expressed in this article are solely those of the authors and do not necessarily represent those of their affiliated organizations, or those of the publisher, the editors and the reviewers. Any product that may be evaluated in this article, or claim that may be made by its manufacturer, is not guaranteed or endorsed by the publisher.
