# Supplementary material for: A Computational Approach to Elucidate the Interactions of Chemicals From Artemisia annua Targeted Toward SARS-CoV-2 Main Protease Inhibition for COVID-19 Treatment
Source: Front Med (Lausanne). 2022 Jun 15;9:907583. doi: 10.3389/fmed.2022.907583 (PMC9240657; doi:10.3389/fmed.2022.907583)
Supplement: Supplementary file 1 [file Table_1.DOCX]

Supplementary Material

**Table S1: The docking scores (kcal/mol) and binding free energy (Δ*G*_bind_) MM-GBSA of 168 phytochemical constituents of *Artemisia annua* against SARS-CoV-2 main protease**

| Serial Number | PubChem CID | docking score | **Δ**G Bind | **Δ**G Bind Coulomb | **Δ**G Bind Covalent | **Δ**G Bind Hbond | **Δ**G Bind Lipo | **Δ**G Bind Packing | **Δ**G Bind vdW |
| --- | --- | --- | --- | --- | --- | --- | --- | --- | --- |
|  | 5320946 | -7.835 | -49.53 | -14.04 | 4.05 | -1.48 | -7.8 | -4.39 | 12.69 |
|  | 5280862 | -7.813 | -45.34 | -11.95 | 4.72 | -1.35 | -6.27 | -4.66 | 14.89 |
|  | 5280863 | -7.648 | -42.09 | -12.94 | 3.53 | -1.36 | -5.68 | -4.83 | 16.46 |
|  | 5282160 | -7.548 | -47.93 | -35.28 | 5.34 | -4.07 | -9.88 | -3.31 | 35.39 |
|  | 5280443 | -7.489 | -39.67 | -9.98 | 4.48 | -1.35 | -5.09 | -4.88 | 13.62 |
|  | 5320462 | -7.383 | -46.7 | -24.39 | 3.1 | -1.73 | -11.02 | -3.18 | 25.17 |
|  | 5280804 | -7.331 | -44.77 | -9.6 | 2.05 | -2.87 | -10.85 | -2.67 | 28.01 |
|  | 5282102 | -7.226 | -50.61 | -11.26 | 2.98 | -2.26 | -11.52 | -2.54 | 23.26 |
|  | 5280637 | -7.161 | -44.92 | -36.17 | 5.52 | -2.87 | -9.78 | -2.85 | 37.88 |
|  | 5281654 | -7.153 | -44.74 | -13.02 | 3.87 | -1.38 | -6.31 | -4.74 | 17.57 |
|  | 5280445 | -7.133 | -40.12 | -14.13 | 0.77 | -2.57 | -4.73 | -3.58 | 16.17 |
|  | 5280699 | -7.105 | -45.85 | -27.6 | 3.75 | -1.82 | -11.16 | -3.09 | 29.66 |
|  | 5280343 | -7.037 | -41.32 | -12.7 | 3.72 | -1.37 | -5.6 | -4.76 | 17.93 |
|  | 5281691 | -7.016 | -52.39 | -24.72 | 2.76 | -1.93 | -9.93 | -4.33 | 20.26 |
|  | 5281608 | -6.978 | -52.7 | -25.51 | 1.52 | -1.81 | -10.96 | -3.16 | 28.09 |
|  | 5748611 | -6.955 | -44.36 | -25.85 | 7.25 | -2.31 | -9.78 | -3.87 | 28.99 |
|  | 5379563 | -6.923 | -47.58 | -13.68 | 1.69 | -1.74 | -10.69 | -3.14 | 20.52 |
|  | 5281678 | -6.857 | -45.44 | -25.04 | 2.75 | -2.38 | -8.28 | -4.33 | 34.25 |
|  | 74208821 | -6.801 | -64.74 | -49.74 | 2.81 | -4.05 | -11.45 | -2.92 | 44.93 |
|  | 5318214 | -6.772 | -43.55 | -7.34 | 3.37 | -1.81 | -6.87 | -3.69 | 11.62 |
|  | 5315263 | -6.758 | -41.61 | -17.55 | 7.73 | -1.94 | -11.77 | -3.13 | 27.15 |
|  | 5320351 | -6.745 | -49.34 | -22 | 8.4 | -1.71 | -11.25 | -2.48 | 28.21 |
|  | 5316832 | -6.664 | -51.96 | -11.89 | 8.37 | -1.45 | -10.86 | -3.96 | 12.89 |
|  | 11230 | -6.627 | -26.49 | -12.77 | 1.98 | -0.49 | -12.18 | 0 | 9.28 |
|  | 5377945 | -6.627 | -44.15 | -17.41 | 1.99 | -1.56 | -8.53 | -4.1 | 27.3 |
|  | 162464 | -6.626 | -41.84 | -24.51 | 3.98 | -1.7 | -9.5 | -3.01 | 28.6 |
|  | 160237 | -6.583 | -42.75 | -14.39 | 0.5 | -1.76 | -8.72 | -3.74 | 20.75 |
|  | 5280666 | -6.54 | -38.11 | -21.44 | 1.8 | -1.32 | -7.31 | -3.06 | 26.41 |
|  | 97214 | -6.462 | -33.65 | -16.54 | 6.31 | -1.79 | -9.17 | -3.13 | 27.67 |
|  | 44259809 | -6.405 | -57.52 | -30.15 | 1.85 | -3 | -10.92 | -3.14 | 33.31 |
|  | 5280460 | -6.377 | -40.54 | -8.76 | 0.65 | -1.18 | -9.1 | -3.05 | 9.11 |
|  | 21632759 | -6.271 | -29.43 | -5.12 | 1.09 | -0.62 | -10.82 | 0 | 10.13 |
|  | 12046149 | -6.243 | -36.04 | -6.61 | 1.96 | -0.5 | -13.91 | 0 | 13.06 |
|  | 10364 | -6.188 | -36.09 | -7.78 | 0.94 | -0.57 | -11.26 | -1.25 | 9.64 |
|  | 5281603 | -6.182 | -40.73 | -15.37 | 3.07 | -1.55 | -8.81 | -4.33 | 30.36 |
|  | 326 | -6.039 | -33.77 | -3.22 | 0.41 | -0.27 | -11.01 | -1.09 | 7.1 |
|  | 10657 | -6.001 | -34.39 | -1.98 | 1.38 | 0 | -13.78 | 0 | 9.77 |
|  | 94266 | -5.996 | -33.51 | -8.15 | 2.06 | -0.61 | -6.76 | 0 | 10.83 |
|  | 6989 | -5.971 | -35.7 | -12.53 | 3.75 | -0.62 | -11.31 | -1.18 | 11.53 |
|  | 8417 | -5.952 | -35.95 | -15.61 | 1.14 | -1.01 | -7.77 | -2.15 | 18.14 |
|  | 853433 | -5.902 | -39.08 | -10.48 | 0.81 | -1.09 | -11.45 | -1.3 | 7.1 |
|  | 10398656 | -5.881 | -37.74 | -5.09 | 0.98 | -0.01 | -15.06 | 0 | 14.63 |
|  | 65030 | -5.88 | -29.27 | -15.37 | 1.76 | -1.57 | -8.56 | 0 | 24.59 |
|  | 439514 | -5.843 | -37.22 | -18.29 | 5.15 | -2.32 | -10.97 | -4.47 | 34.05 |
|  | 93081 | -5.811 | -32.19 | -3.11 | 0.89 | 0 | -11.55 | 0 | 11.69 |
|  | 12306048 | -5.773 | -36.68 | -1.29 | 0.44 | 0 | -14.91 | 0 | 10.32 |
|  | 102553 | -5.753 | -27.7 | -6.68 | -0.16 | -0.59 | -4.95 | 0 | 10.83 |
|  | 20197 | -5.748 | -30.97 | -3.02 | -0.52 | 0 | -11.97 | -1.2 | 11.02 |
|  | 5280863 | -5.742 | -25.59 | -23.11 | 2.97 | -1.38 | -5.58 | -4.95 | 42.93 |
|  | 17868 | -5.741 | -22.32 | -1.14 | 3.78 | 0 | -13.14 | 0 | 7.75 |
|  | 10703 | -5.723 | -31.11 | -2.2 | 0.48 | 0 | -11.61 | -1.07 | 9.31 |
|  | 7461 | -5.704 | -29.81 | -3.24 | 0.29 | 0 | -11.22 | 0 | 9.2 |
|  | 441005 | -5.699 | -36.58 | -1.58 | 0.86 | 0 | -14.81 | 0 | 10.47 |
|  | 5280443 | -5.693 | -26.45 | -39.37 | 1.61 | -1.77 | -6.5 | -3.19 | 52.49 |
|  | 5377945 | -5.675 | -33.02 | -40.37 | 0.07 | -2.32 | -8.43 | -3.36 | 50.22 |
|  | 5280862 | -5.673 | -24.52 | -26.97 | 3.65 | -1.31 | -5.81 | -5.06 | 48.63 |
|  | 7462 | -5.656 | -28.16 | -2.44 | 3.88 | 0 | -11.42 | 0 | 8.23 |
|  | 11000442 | -5.593 | -34.76 | -11.26 | 0.06 | -1.16 | -8.36 | 0 | 18.31 |
|  | 162222210 | -5.59 | -30.74 | -2.67 | -0.15 | 0 | -11.61 | -1.18 | 9.17 |
|  | 7463 | -5.575 | -30.24 | -6.8 | 0.21 | -0.06 | -8.27 | -2.57 | 11.59 |
|  | 323 | -5.561 | -19.19 | 18.24 | 5.59 | -1.07 | -8.72 | 0 | -1.9 |
|  | 126891 | -5.495 | -32.07 | -7.66 | 2.73 | -0.66 | -10.2 | 0 | 13.75 |
|  | 12814879 | -5.457 | -34.14 | -10.02 | 0.77 | -1.16 | -8.38 | 0 | 17.3 |
|  | 68827 | -5.443 | -31.61 | -11.03 | 0.62 | -0.62 | -8.06 | 0 | 14.3 |
|  | 636625 | -5.415 | -32.35 | -1.14 | 0.8 | 0 | -10.84 | 0 | 11.05 |
|  | 521334 | -5.4 | -36.9 | -46.02 | 3.41 | -2.98 | -8.29 | -3.14 | 50.57 |
|  | 5281678 | -5.375 | -12.08 | 22.92 | 1.48 | -0.52 | -10.63 | 0 | 6.01 |
|  | 578305 | -5.369 | -24.56 | -0.18 | 5.16 | 0 | -11.88 | 0 | 13.6 |
|  | 5317844 | -5.353 | -31.52 | -12.24 | 0.65 | -0.68 | -8 | 0 | 15 |
|  | 6543478 | -5.296 | -28.84 | -22.64 | 3.32 | -1.39 | -6.22 | -4.87 | 42.88 |
|  | 5281654 | -5.279 | -29.4 | -47.66 | 5.69 | -2.31 | -6.44 | -3.22 | 53.84 |
|  | 5280445 | -5.277 | -42.47 | -4.25 | 4.02 | -1.45 | -8.23 | -4.46 | 10.92 |
|  | 5320946 | -5.236 | -23.35 | -6.82 | 0.51 | -0.54 | -8.53 | 0 | 7.78 |
|  | 527032 | -5.188 | -21.82 | -6.98 | 0.89 | -0.56 | -7.26 | 0 | 7.37 |
|  | 101680 | -5.174 | -30.26 | -1.65 | 2.48 | 0 | -11.94 | 0 | 12 |
|  | 442393 | -5.163 | -26.76 | -2.56 | 1.92 | -0.26 | -11.15 | 0 | 7.5 |
|  | 1254 | -5.141 | -18.44 | -2.55 | 2.62 | 0 | -10.11 | 0 | 9.34 |
|  | 520384 | -5.141 | -25.97 | -41.66 | 2.23 | -1.77 | -6.94 | -3.17 | 57.85 |
|  | 5280666 | -5.131 | -25.01 | -8.85 | 3.53 | -0.66 | -9.07 | 0 | 11.74 |
|  | 42626428 | -5.103 | -26.65 | -2.65 | 0.36 | -0.25 | -8.5 | 0 | 8.22 |
|  | 17100 | -5.062 | -24.11 | -8.33 | 0.77 | -0.61 | -8.32 | 0 | 9.08 |
|  | 102667 | -5.03 | -42.49 | -6.82 | 1.84 | -2.14 | -7.55 | -4.59 | 12.26 |
|  | 5281691 | -5.025 | -32.25 | -38.22 | 0.88 | -1.89 | -10.38 | -3.09 | 59.77 |
|  | 5379563 | -5.012 | -19.75 | -1.64 | 8.07 | 0 | -15.06 | 0 | 10.85 |
|  | 19725 | -5.012 | -19.75 | -1.64 | 8.07 | 0 | -15.06 | 0 | 10.85 |
|  | 6432119 | -5.012 | -25.3 | -7.54 | -0.07 | -0.84 | -7.19 | 0 | 8.47 |
|  | 29025 | -5.004 | -20.95 | -2.45 | 10.06 | 0 | -14.09 | 0 | 9.75 |
|  | 5281515 | -4.98 | -23.32 | -8.6 | 0.38 | -0.59 | -7.28 | 0 | 8.83 |
|  | 64685 | -4.974 | -24.11 | -1.87 | 6.18 | 0 | -13.75 | 0 | 11.42 |
|  | 5281522 | -4.958 | -23.03 | -11.77 | 0.23 | -0.6 | -4.32 | 0 | 11.96 |
|  | 61130 | -4.957 | -11.22 | -1.88 | 3.79 | 0 | -12.07 | 0 | 9.18 |
|  | 6654 | -4.943 | -22.99 | -5.55 | 2.49 | -0.58 | -8.83 | 0 | 6.99 |
|  | 89664 | -4.939 | -35.7 | -0.8 | -0.06 | -0.47 | -11.36 | -2.17 | 8.07 |
|  | 7651 | -4.924 | -25.06 | -22.26 | 3.18 | -1.39 | -5.51 | -4.89 | 43.56 |
|  | 5280343 | -4.907 | -19.73 | -0.95 | 6.39 | 0 | -17.37 | 0 | 11.73 |
|  | 6432312 | -4.892 | -24.67 | -7.93 | 0.19 | -0.88 | -7.86 | 0 | 8.16 |
|  | 10582 | -4.873 | -18.23 | -6.3 | 2.48 | -0.1 | -9.04 | 0 | 10.64 |
|  | 92784 | -4.86 | -17.89 | -3.76 | 2.92 | -0.01 | -11.54 | 0 | 15.15 |
|  | 92231 | -4.847 | -20.32 | -8.3 | 1.81 | -0.83 | -8.68 | 0 | 24.44 |
|  | 14356591 | -4.828 | -35.32 | -7.9 | 2.9 | -0.61 | -13.7 | -2.14 | 21.74 |
|  | 5991 | -4.825 | -23.33 | -9.08 | 0.53 | -0.84 | -7.74 | 0 | 10.52 |
|  | 12315160 | -4.805 | -24.2 | -8.06 | 0.21 | -0.78 | -5.17 | 0 | 8.64 |
|  | 2758 | -4.799 | -19.24 | -1.81 | 1.84 | 0 | -11.73 | 0 | 7.88 |
|  | 101629835 | -4.791 | -18.65 | -3.38 | 4.14 | -0.09 | -10.09 | 0 | 12.6 |
|  | 6427504 | -4.772 | -22.48 | -9.48 | 1.83 | -0.51 | -5.74 | 0 | 12.96 |
|  | 6431301 | -4.769 | -19.54 | -0.51 | 2.31 | 0 | -11.96 | 0 | 8.42 |
|  | 18818 | -4.763 | -23.22 | -7.03 | -0.03 | -1.14 | -6.15 | 0 | 9.67 |
|  | 10545 | -4.751 | -29.75 | -4.72 | 5.92 | -0.41 | -15.09 | 0 | 22.05 |
|  | 92156 | -4.732 | -19.78 | 34.4 | 2.31 | -1.22 | -13.66 | 0 | -8.6 |
|  | 10494 | -4.708 | -31.81 | -9.49 | 3.96 | -0.56 | -13.04 | 0 | 13.89 |
|  | 101679337 | -4.706 | -25.63 | -5.1 | 1.81 | -0.23 | -10.06 | 0 | 10.71 |
|  | 7439 | -4.704 | -19.14 | -1.17 | 6.25 | 0 | -12.42 | 0 | 11.6 |
|  | 21775138 | -4.655 | -24.29 | -2.59 | 0.9 | -0.43 | -7.17 | 0 | 10.16 |
|  | 10899521 | -4.649 | -17.72 | -8.74 | 0.49 | -0.58 | -3.6 | 0 | 12.15 |
|  | 2537 | -4.64 | -21.99 | -4.99 | -0.2 | -0.54 | -7.64 | 0 | 7.97 |
|  | 88302 | -4.626 | -15.72 | -0.58 | 2.76 | -0.19 | -10.49 | 0 | 14.48 |
|  | 1742210 | -4.614 | -21.78 | 11.75 | 2.02 | -2 | -7.48 | 0 | 2.61 |
|  | 10922465 | -4.596 | -26.5 | -25.18 | 3.02 | -1.51 | -9.21 | -4.27 | 47.61 |
|  | 5281603 | -4.596 | -29.92 | -10.92 | 3.08 | -0.74 | -11.48 | 0 | 10.39 |
|  | 5352516 | -4.589 | -19.99 | 0.24 | 0.3 | 0 | -8.25 | 0 | 7.08 |
|  | 14896 | -4.583 | -19.45 | -3.91 | 1.24 | -0.52 | -5.65 | 0 | 10.06 |
|  | 84532 | -4.543 | -13.48 | -0.99 | 4.68 | 0 | -12.45 | 0 | 8.79 |
|  | 28930 | -4.529 | -34.69 | -4.81 | 4.05 | -0.6 | -17.33 | 0 | 24.57 |
|  | 5280794 | -4.519 | -37.96 | -4.27 | 0.33 | 0 | -12.75 | -1.98 | 9.07 |
|  | 3083613 | -4.457 | -33.07 | -14.46 | 2.85 | -1.13 | -8.71 | 0 | 11.77 |
|  | 11389649 | -4.455 | -34.3 | -12.65 | 1.99 | -1.03 | -11.62 | 0 | 11.55 |
|  | 5315406 | -4.385 | -35.51 | -6.28 | 1.85 | -0.54 | -12.34 | 0 | 8.65 |
|  | 10586 | -4.375 | -44.03 | -11.99 | 0.47 | -0.54 | -13.07 | 0 | 13.11 |
|  | 102368592 | -4.362 | -34.18 | -2.31 | 0.32 | -0.12 | -14.8 | 0 | 21.1 |
|  | 73170 | -4.306 | -40.88 | -19.19 | 2.48 | -2.16 | -10.92 | -3.34 | 34.61 |
|  | 5282102 | -4.284 | -24.53 | -5.3 | 6.87 | -0.16 | -13.01 | 0 | 12.92 |
|  | 524254 | -4.261 | -11.45 | -1.89 | 1.85 | 0 | -6.53 | 0 | 14.13 |
|  | 90658163 | -4.244 | -15.92 | -1.37 | 6.28 | 0 | -14.89 | 0 | 11.49 |
|  | 6918391 | -4.164 | -26.9 | -9.23 | 2.52 | -0.34 | -13.28 | 0 | 12.66 |
|  | 12308602 | -4.163 | -13.1 | -2.37 | 2.46 | 0 | -8.41 | 0 | 8.86 |
|  | 6616 | -4.161 | -33.21 | -45.71 | 4.89 | -4.52 | -9.91 | -2.5 | 62.15 |
|  | 5280804 | -4.158 | -27.47 | -4.51 | 1.41 | -0.06 | -12.25 | 0 | 10.83 |
|  | 68346 | -4.13 | -25.48 | -1.45 | 1.08 | 0 | -10.65 | 0 | 8.21 |
|  | 22311 | -4.113 | -20.26 | -0.41 | 0.29 | 0 | -9.4 | 0 | 9.44 |
|  | 44567200 | -4.089 | -33.8 | -2.2 | 1.07 | -0.12 | -15.48 | 0 | 21.4 |
|  | 73145 | -4.086 | -34.09 | -2.53 | 1.61 | -0.07 | -13.81 | 0 | 19.5 |
|  | 111220 | -4.069 | -23.77 | 0.51 | 1.14 | 0 | -10.22 | 0 | 8.89 |
|  | 5281520 | -4.052 | -17.13 | -0.58 | 0.56 | 0 | -7.85 | 0 | 14.61 |
|  | 91723653 | -4.016 | -22.3 | -5.9 | 6.03 | -0.54 | -13.3 | 0 | 12.46 |
|  | 637566 | -3.953 | -20.08 | -0.07 | 0.08 | 0 | -9.63 | 0 | 10.39 |
|  | 42608158 | -3.934 | -34.25 | -2.89 | 0.78 | -0.12 | -14.12 | 0 | 20.79 |
|  | 12306155 | -3.908 | -25.73 | -6.51 | 3.11 | -0.55 | -11.64 | 0 | 10.96 |
|  | 14485465 | -3.893 | -24.08 | -9.75 | 2.02 | -0.55 | -8.07 | 0 | 11.53 |
|  | 162222210 | -3.832 | -34.27 | -9.34 | 3.42 | -0.47 | -8.7 | 0 | 11.21 |
|  | 6549 | -3.83 | -35.83 | -9.24 | 8.01 | -1.6 | -17.38 | 0 | 18.88 |
|  | 5370101 | -3.791 | -17.19 | -0.9 | 0.32 | 0 | -6 | 0 | 7.36 |
|  | 1549025 | -3.782 | -30.52 | -1.3 | 0.97 | -0.18 | -12.07 | 0 | 18.05 |
|  | 5205968 | -3.774 | -27.54 | -4.34 | 3.08 | -0.44 | -12.43 | 0 | 8.04 |
|  | 124349839 | -3.658 | -23.8 | -1.97 | 2.3 | 0 | -14.23 | 0 | 8.67 |
|  | 6448 | -3.623 | -31.92 | -2.8 | 1.76 | 0 | -13.38 | 0 | 8.07 |
|  | 119242 | -3.596 | -25.47 | -3.37 | 2.83 | 0 | -13.1 | 0 | 8.17 |
|  | 6538431 | -3.576 | -29.33 | -3.36 | 0.77 | 0 | -11.06 | 0 | 17.82 |
|  | 5281553 | -3.485 | -31.12 | -6.25 | 3.61 | -0.65 | -12.29 | 0 | 11.69 |
|  | 5320377 | -3.37 | -12.77 | -17.63 | 0.55 | -0.5 | -3.81 | -4.79 | 43.38 |
|  | 519872 | -3.349 | -30.47 | -7.44 | 2.11 | -0.63 | -13.37 | 0 | 22.2 |
|  | 91472 | -3.318 | -24.01 | -2.8 | 7.94 | 0 | -13.69 | 0 | 9.1 |
|  | 10975 | -3.177 | -30.15 | -6.31 | 3.42 | -1.44 | -9.47 | 0 | 12.38 |
|  | 5280460 | -2.877 | -18.29 | -6.28 | 0.21 | -0.44 | -1.66 | 0 | 4.37 |
|  | 222284 | -2.779 | -32.41 | -14.71 | 0.62 | -1.53 | -12.49 | 0 | 22.67 |
|  | 68406 | -2.751 | -24.13 | -2.61 | 0.87 | 0 | -11.46 | 0 | 9.19 |
|  | 31253 | -2.641 | -35.79 | -3.61 | 1.68 | -0.45 | -14.44 | 0 | 14.51 |
|  | 126891 | -2.637 | -33.31 | -2.1 | 3.3 | 0 | -17.37 | 0 | 10.02 |
|  | 6584 | -2.45 | -35.72 | -10.48 | 3.25 | -0.56 | -15.14 | 0 | 18.25 |
